# Successful 6-year follow-up of treatment for IgA nephropathy complicated by IgA-λ monoclonal gammopathy with multi-system damage: a case report

**DOI:** 10.3389/fimmu.2026.1815188

**Published:** 2026-05-29

**Authors:** Yingying Li, Xiuxiu Li, Ying Lu, Meichun Huang

**Affiliations:** 1 Renal Department, Tongde Hospital of Zhejiang Province Affiliated to Zhejiang Chinese Medical University (College of Integrated Traditional Chinese and Western Medicine Clinical Medicine), Hangzhou, China; 2 Renal Department, Tongde Hospital of Zhejiang Province Affiliated to Zhejiang Chinese Medical University, Hangzhou, China

**Keywords:** IgA nephropathy, kidney transplantation, monoclonal gammopathy, monoclonal gammopathy of renal significance (MGRS), monoclonal gammopathy of rheumatologic significance (MGRhS)

## Abstract

Immunoglobulin A nephropathy (IgAN) is the most common primary glomerular disease in China and a major contributor to the need for renal transplantation. IgAN is characterized by the mesangial deposition of IgA-dominant or IgA-specific immune complexes, accompanied by complement components, and is recognized as a polyclonal autoimmune disorder largely restricted to the kidneys. But approximately 9.2% of patients with IgAN demonstrate evidence of monoclonal IgA deposition, making IgAN with monoclonal IgA deposition and related nephropathies clinically challenging conditions. Our report describes a rare case of IgAN complicated by IgA-λ monoclonal gammopathy of rheumatologic significance(MGRhS). Although the renal pathology in the patient did not suggest monoclonal gammopathy of renal significance (MGRS), the patient showed a poor initial response to corticosteroid, immunosuppressive therapy, and rituximab, followed by progressive clinical deterioration with multi-system damage, including persistent fever, normocytic anemia, recurrent hemoptysis, and sensorimotor peripheral neuropathy. Clone-directed treatment targeting the underlying plasma cell disorder led to marked clinical improvement. The patient subsequently underwent kidney transplantation with favorable outcomes after three years. Over a three-year follow-up period after transplantation, graft function remained stable and no evidence of disease recurrence was detected. In conclusion, when renal pathology in IgAN does not fulfill diagnostic criteria for MGRS, careful evaluation for IgA monoclonal gammopathy is warranted. It may represent MGRhS associated with multisystem involvement. Complete elimination of the monoclonal protein before transplantation is critical for achieving sustained allograft survival.

## 1 Introduction

IgA nephropathy (IgAN) represents the most prevalent primary glomerular disease in China, with nearly 40% of affected individuals progressing to end-stage renal disease (ESRD) within two decades of diagnosis, making a leading population requiring renal transplantation. IgAN is characterized by mesangial deposition of IgA-dominant or IgA-specific immune complexes and is a polyclonal autoimmune disorder primarily confined to the kidneys (1, 2). Recent data suggest that approximately 9.2% of patients diagnosed with IgAN show evidence of monoclonal IgA deposition (3). Rports addressing this condition remain limited, particularly in the context of renal transplantation. When patients with monoclonal gammopathy of renal significance(MGRS) are overlooked, the likelihood of post-transplant disease recurrence is significantly increased (4). In our report, a sporadic case of successful treatment in IgAN complicated by IgA-λ monoclonal gammopathy with multi-system damage is presented. The patient’s clinical symptoms had significantly improved. Subsequent 6-year follow-up confirmed successful treatment with normal graft function and an overall healthy condition.

## 2 Case presentation

**Figure 1:**
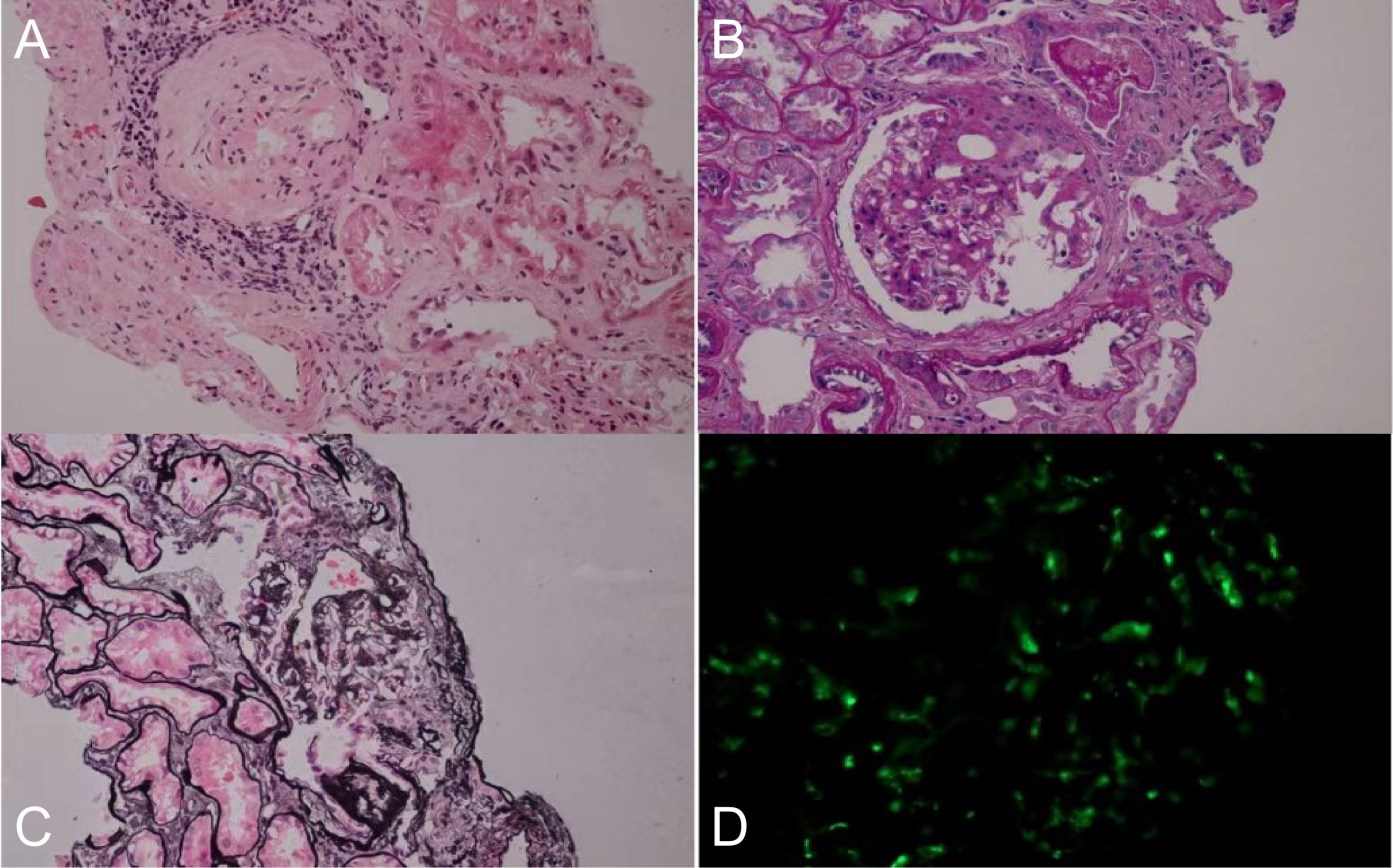
Pathological findings in a renal biopsy specimen. **(A)** HE staining reveals a total of 14 glomeruli, among which 5 show global sclerosis. **(B)** PAS staining demonstrates proliferation of mesangial cells and matrix. **(C)** PASM staining displays patent capillary loops without significant thickening of the basement membrane. **(D)** Immunofluorescence results indicate the presence of IgA deposition.

A 50-year-old Chinese male was admitted in November 2018 for initiation of hemodialysis, with pathologically confirmed immunoglobulin A nephropathy (IgAN) for two years and clinical deterioration over the preceding eight months. In 2012, the patient had a serum creatinine (SCr) of 123 μmol/L (40-83 μmol/L), urinary total protein(UTP) 0.38 g/24h(0-0.2g), and a peak blood pressure of 167/98 mmHg. In May 2016, because disease progression was evident with increased UTP to 1.54 g and SCr of 117 μmol/L, The performed renal biopsy revealed focal proliferative sclerosing IgAN (Lee’s grade IV; Oxford classification M1E0S0T1) (**Figure 1**). Among 14 glomeruli examined, 5 showed global sclerosis, and 2 demonstrated periglomerular fibrosis. The remaining glomeruli displayed mild to moderate mesangial cell and matrix proliferation with focal segmental predominance. Tubular epithelial cells displayed vacuolar and granular degeneration with multifocal tubular atrophy involving approximately 30% of the cortex. The interstitium showed multifocal inflammatory cell infiltration with fibrosis, and small arteries showed wall thickening with luminal narrowing. Immunofluorescence revealed IgG−, IgM+, IgA++, and C3+ staining, while C1q, HBsAg, HBcAg, HBeAg, IgG1, IgG4, and PLA2R were negative; both κ and λ light chains were positive. Albumin (ALB) staining revealed protein resorption droplets within tubular epithelial cells. Special stains, including Congo red and oxidized Congo red, were negative. Electron microscopy of one glomerulus demonstrated marked vacuolar degeneration of capillary endothelial and parietal epithelial cells, swollen podocytes with extensive foot process effacement, mesangial hypercellularity with sparse electron-dense deposits, glomerular basement membrane (GBM) thickness ranging from 250 to 460 nm, tubular atrophy, interstitial lymphocytic and monocytic infiltration with collagen fiber proliferation, and small arterial wall thickening. The renal biopsy was consistent with proliferative glomerulonephritis with predominant IgA deposition. Both κ and λ light chains were positive without evidence of light chain restriction. Morphological and ultrastructural findings were not suggestive of monoclonal immunoglobulin deposition disease (MIDD), as no mesangial nodules or fine granular electron-dense deposits were observed. Congo red staining excluded amyloidosis. The findings were diagnostic of primary IgA nephropathy.

750 mg/day mycophenolate mofetil and 16 mg/day methylprednisolone were administered, but discontinued after two months due to severe pulmonary infection, UTP 2.3g, SCr117μmol/L. Thereafter, the patient’s condition remained relatively stable with blockade of the renin-angiotensin-aldosterone system. By May 2018, Urine analysis showed red blood cell +++/HP, protein +++, and UTP 5.16g. Serum analysis revealed ALB 31 g/L, SCr 133μmol/L. A subsequent regimen of prednisone (50 mg/day) combined with mycophenolate mofetil (100 mg/day) was ineffective, with UTP escalating to 13.8 g and SCr rising to 297 µmol/L. In October 2018, the patient received high-dose intravenous immunoglobulin (10 g/day for 7 days) combined with rituximab (300 mg). By November 2018, UTP was 6.05 g, and SCr was 420 μmol/L, indicating progression to ESRD.

The patient was hospitalized on November 28, 2018, and initiated on thrice-weekly hemodialysis. Past medical history included well-controlled hypertension and diabetes. On admission, vital signs were: temperature 37.1 °C, pulse 90 beats/min, respiration 16 breaths/min, and blood pressure 133/90 mmHg. The patient appeared pale, without jaundice or petechiae. No superficial lymphadenopathy was palpable. Cardiopulmonary examination was unremarkable. The abdomen was soft and non-tender, with no hepatosplenomegaly. Bilateral lower extremity edema was present. Laboratory evaluation revealed ALB of 28.1 g/L, UTP of 4.98 mg, hematuria +++/Hp(1039/ul), SCr of 527 μmol/L, hemoglobin of 63 g/L, and a B-cell proportion of 0.1%. Serum phosphorus was 1.38 mmol/L (0.83-1.48 mmol/L). The patient was diagnosed with rapidly progressive glomerulonephritis secondary to previously confirmed IgAN.

A systematic differential diagnosis was performed to determine the cause of the decline in renal function. Serum cryoglobulins were negative, and the patient had no purpura, arthralgia, or peripheral neuropathy, ruling out cryoglobulinemia. ANA and anti-dsDNA were negative, with no malar rash or oral ulcers, excluding systemic lupus erythematosus (SLE). Anti-neutrophil cytoplasmic antibodies (ANCA) were negative, excluding ANCA-associated vasculitis. The patient did not develop skin purpura, arthralgia, or abdominal pain throughout the disease course. Lack of response to immunosuppressive therapy further excluded IgA vasculitis. However, due to bilateral kidney atrophy and the high risk of bleeding, the patient and his family declined repeat renal biopsy. Given the potential for an underlying hematologic disorder, further evaluation was conducted to assess clonal plasma cell or B-cell disease. Therefore, on December 4,further investigations revealed monoclonal IgA-λ on serum and urine immunofixation electrophoresis (IFE). Bone marrow aspiration showed that plasma cells accounted for 3% of all nucleated cells. Flow cytometry analysis identified an abnormal clonal plasma cell population accounting for 0.7% of cells. Skeletal imaging, including plain radiographs of the pelvis, skull (anteroposterior and lateral views), and lumbar spine, showed no lytic lesions or abnormal findings. According to established diagnostic criteria for multiple myeloma (5), ≥ 10% clonal plasma cells in bone marrow or a biopsy-proven plasmacytoma is required; this patient did not meet these criteria. A diagnosis of IgA nephropathy complicated by IgA-λ monoclonal gammopathy was therefore established, and maintenance dialysis was continued.

Two weeks later (December 13), the patient’s clinical status deteriorated, presenting with low-grade fever, cough with dark-brown hemoptysis, gross hematuria, and reflex vasovagal syncope. Laboratory evaluation revealed hemoglobin 57 g/L, ALB 22.3 g/L, SCr 701 μmol/L, C-reactive protein 12.08 mg/L, and procalcitonin 0.37 ng/mL. Urine analysis showed 1152 red blood cells/μL and 9.47 mg of total urinary protein. Comprehensive virological assays (HBV, HCV, HIV, CMV, EBV), pathogen nucleic acid testing, and bacterial and fungal cultures of blood and sputum were negative. Chest CT demonstrated diffuse bilateral exudative changes without mass lesions, nodules, cavitations, or mediastinal lymphadenopathy, suggesting pulmonary inflammation. Empirical antimicrobial therapy with piperacillin-tazobactam and caspofungin was initiated, but pulmonary lesions persisted. The patient continued to have low-grade fever and hemoptoic sputum. Further bronchoscopy with bronchoalveolar lavage fluid (BALF) analysis excluded bacterial, fungal, mycobacterial, and viral infections (including CMV and EBV). Cytology and flow cytometry detected no malignant cells, effectively ruling out active malignancy. Despite ongoing dialysis, the patient showed progressive multisystem dysfunction not fully attributable to IgA nephropathy. Key laboratory findings are summarized in **Table 1** and **Table 2**. A clinical diagnosis of systemic multiorgan injury associated with monoclonal IgA-λ gammopathy (MGRS) was established.

**Table 1:** Laboratory characteristics at baseline and post- Bortezomib therapy.

Component	Baseline	Bortezomib therapy (1 month)	Bortezomib therapy (2 months)	Reference range
Urinalysis				
Urinary protein	2+	3+	+	Negative
Red blood Cells (n/HPF)	3+	+	–	0-3
White blood Cells (n/HPF)	–	–	–	0-5
Urinary protein excretion (g/24 h)	4.98	5.78	3.42	0-0.2
Hematology				
White blood cells (×10^9^ /L)	5.2	10.6	6.5	3.5-9.5
Neutrophils ratio(%)	72.3	85.2	80.6	40-75
Eosinophils (×10^9^/L)	0.19	0.02	0.08	0.02-0.52
Eosinophil ratio (%)	3.6	0.2	1.2	0.4-8
Platelets (×10^9^ /L)	119	63	147	125-350
Hemoglobin (g/L)	57	89	101	115-150
C-reactive protein (mg/L)	12.08	1.1	0.4	0-8
Blood chemistry				
BUN (mmol/L)	24.3	16.7	33.1	2.6-7.5
Serum creatinine (µmol/L)	574	388	415	40-83
Serum albumin (g/L)	22.3	30.4	34.5	40-55
Uric acid (µmol/L)	274	257	476	140-340
Blood immunology				
C3(g/L)	0.67	0.63	0.62	0.70-1.40
C4(g/L)	0.19	0.18	0.17	0.10-0.40
IgG(g/L)	2.3	5.66	4.99	8.60-17.40
IgA(g/L)	3.25	0.25	0.29	1.00-4.20
IgM(g/L)	0.33	0.18	0.18	0.50-2.80
MPO-ANCA	Negative	Negative	Negative	Negative
P-ANCA	Negative	Negative	Negative	Negative
PR3-ANCA	Negative	Negative	Negative	Negative
C-ANCA	Negative	Negative	Negative	Negative
Anti-GBM antibody (RU/mL)	Negative	Negative	Negative	<20
ANA	Negative	Negative	Negative	Negative
Coombs test	Negative	Negative	Negative	Negative
SIFE	Positive	Negative	Negative	Negative

HPF, high power field; BUN, Blood urea nitrogen; MPO-ANCA, myeloperoxidase-anti-neutrophil cytoplasmic antibodies; PR3-ANCA, proteinase-3-anti-neutrophil cytoplasmic antibodies; p-ANCA, Anti-myeloperoxidase ANCA; c-ANCA, Anti-proteinase-3 ANCA; anti-GBM, anti-glomerular basement membrane; ANA, antinuclear antibodies; C3, complement 3; C4, complement 4; SIFE, serum immunofixation electrophoresis.

**Table 2:** Clinical timeline from initial diagnosis to post-transplant follow-up.

Time point	Event	Key laboratory data	Intervention
2012	CKD	UTP 0.38 g , SCr 123 μmol/L	RAAS blockade
May 2016	Diagnosis of IgAN (Lee grade IV, M1E0S0T1)	UTP 1.54 g, SCr 117 μmol/L	CS+ MMF
May 2018	IgAN with rapidly progressive glomerulonephritis (RPGN)	UTP 5.16 g, SCr 133 μmol/L	CS+MMF+RTX
Dec 2018	Diagnosis of IgAN with MG, pulmonary hemorrhage, anemia	Hematuria +++/HPF (1152/μL), UTP 9.47 g, Hb 57 g/L, Alb 22.3 g/L, SCr701 μmol/L, CRP 12.08 mg/L, PCT 0.37 ng/mL; serum and urine immunofixation electrophoresis positive for monoclonal IgA-λ	antibiotics+albumin support+HD tiw
Jan 2019	Diagnosis of IgAN with MGRhS	Urine output 1500 mL/day, Hematuria 42/μL, UTP 5.78 g, Hb 89 g/L, SCr 388 μmol/L	Bortezomib + dexamethasone+HD tiw
Mar 2019	Favorable treatment response	UTP 3.42g, Hb 108 g/L, SCr 415 μmol/L; immunofixation electrophoresis turned negative	Consolidation chemotherapy+HD biw
Dec 2019	Sustained remission	Hematuria 16/μL, UTP 0.39 g, Hb 101 g/L, SCr 785 μmol/L; serum and urine immunofixation electrophoresis negative	Discontinued anti-plasma cell therapy+HD biw
Apr 2022	Successful kidney transplantation	Serum and urine immunofixation electrophoresis negative for >36 months	kidney transplantation
Dec 2025	Three-year post-transplant follow-up	SCr100 μmol/L; serum and urine immunofixation electrophoresis negative; urinalysis normal	low-dose prednisone with tacrolimus and MMF

CKD, Chronic Kidney Disease; MG, Monoclonal Gammopathy; HPF, high power field; UTP,24-hour Urinary Total Protein; SCr,Serum Creatinine;Hb,Hemoglobin; CS, Corticosteroid; RTX, rituximab; MMF,Mycophenolate Mofetil; HD, Hemodialysis; tiw, three times a week; biw, twice a week.

**Figure 2:**
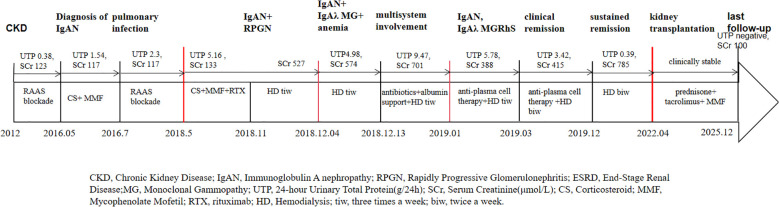
Clinical timeline from initial diagnosis to post-transplant follow-up.

On January 8, 2019, the patient commenced a bortezomib-dexamethasone regimen, comprising bortezomib 2.4 mg subcutaneously on days 1, 4, 8, and 11, and dexamethasone 20 mg on days 1–2, 4–5, 8–9, and 11–12. The first treatment cycle elicited rapid clinical improvement: hemoptysis and gross hematuria resolved, daily urine output increased from 500 mL to 1500 mL, and diffuse pulmonary lesions fully resolved. Hemoglobin rose to 89 g/L, C-reactive protein normalized, urinary red blood cells decreased to 42/μL, SCr declined to 388 μmol/L and UTP 5.78g. Dialysis frequency was reduced from three to two sessions per week. After three cycles, both serum and urine IFE became negative in March, and the serum free light chain ratio normalized; however, SCr remained elevated at 415 μmol/L and UTP was 3.42g. The treatment was then transitioned to oral ixazomib, which was continued until December 9, 2019, at which point anti-plasma cell therapy was discontinued. At that time, UTP was 0.39 g, and SCr was 785 μmol/L. Follow-up every three months demonstrated persistent normalization of immunofixation and free light chain results. In April 2022, the patient underwent successful kidney transplantation at an external center and was liberated from dialysis post-transplant. Postoperative management included long-term triple immunosuppressive therapy with tacrolimus, mycophenolate mofetil, and prednisone, with prednisone tapered from 15 mg/day to a maintenance dose of 2.5 mg/day. At the most recent follow-up in December 2025, allograft function remained normal (SCr 100 µmol/L), with a negative urine analysis and no evidence of monoclonal gammopathy recurrence. The treatment workflow is shown in **Figure 2**.

## 3 Discussion

Monoclonal gammopathy encompasses a spectrum of disorders defined by the clonal expansion of B cells or plasma cells that secrete monoclonal immunoglobulins (6). These disorders are classified based on clinical manifestations and fulfillment of hematologic malignancy criteria. Monoclonal gammopathy of undetermined significance (MGUS) represents an asymptomatic, benign clonal condition without end-organ injury, requiring no active treatment but periodic monitoring for progression risk (7). Monoclonal gammopathy of clinical significance (MGCS) refers to conditions in which monoclonal immunoglobulins cause organ dysfunction or immune perturbations without meeting the criteria for a hematologic malignancy. Typically driven by a small clonal population, MGCS pathophysiology involves mechanisms such as monoclonal immunoglobulin deposition, autoantibody activity, complement activation, and cytokine dysregulation. The affected organ system, including renal, neurological, and cutaneous subtypes, further categorizes MGCS (8). Although monoclonal gammopathy has been classically linked to hematologic malignancies, its involvement in autoimmune and rheumatologic conditions has received increasing attention in recent years.

In 2012, the International Kidney and Monoclonal Gammopathy Research Group (IKMG) introduced the concept of monoclonal gammopathy of renal significance (MGRS), with diagnostic criteria further refined in 2017 (9). In its most recent update, the IKMG redefined MGRS as a group of disorders characterized by renal injury caused by MIg or their fragments from a clonal plasma cell or B cell proliferative process, which do not meet the diagnostic criteria for multiple myeloma or other hematologic malignancies (10).Extending this conceptual framework, Quartuccio et al. proposed in 2025 the term monoclonal gammopathy of rheumatologic significance (MGRhS), describing non-malignant or pre-malignant systemic condition related to a monoclonal immunoglobulin and clonal B cells, capable of producing multi-organ damage or influencing the therapeutic management of rheumatologic diseases (11).

The conceptual understanding of monoclonal gammopathy continues to evolve, particularly in cases where IgA nephropathy is complicated by IgA monoclonal gammopathy, which presents more complex clinical and pathological mechanisms. Reports on this condition remain scarce.

In IgAN, IgA exists as monomers or polymers. Glomerular deposits predominantly consist of pIgA derived from mucosa-associated lymphoid tissue–activated plasma cells. Compared with monomeric IgA, pIgA shows larger molecular size, slower clearance, higher mesangial affinity, and greater complement activation, increasing its tendency for mesangial and small-vessel deposition (1, 12). Approximately 9.2% of IgAN patients show monoclonal IgA deposition (3), suggesting a potential underlying clonal disorder in a subset of cases. Monoclonal IgA-λ deposition is mediated through two mechanisms: overproduction of Gd-IgA1-λ by mucosal B cells, and the high affinity of negatively charged λ light chains for mesangial structures, promoting selective mesangial accumulation (13, 14). These mechanisms predominantly pertain to primary IgAN, whereas MGRS-related monoclonal IgA deposition originates from clonal plasma or B-cell proliferation, with distinct pathophysiological characteristics between the two processes.

IgA-λ monoclonal gammopathy represents a rare subtype within the MGRS spectrum. Its pathological hallmark is the production of monoclonal IgA-λ and its fragments by clonal plasma cells, which may deposit directly in renal tissue as nephrotoxic agents or mediate organ damage through indirect immune mechanisms. The IgA-λ subtype demonstrates greater clinical heterogeneity and poses higher diagnostic complexity. The heterogeneity of IgA-λ MGRS is reflected first in its diverse pathological phenotypes. It may present as classic primary IgA nephropathy, characterized by mesangial proliferation with predominantly mesangial IgA deposition, or as Proliferative Glomerulonephritis with Monoclonal Immunoglobulin Deposits (PGNMID), and in rarer instances, as crystalline deposits or fibrillary glomerulonephritis–like patterns (15, 16). This morphological diversity corresponds to a wide spectrum of clinical presentations, ranging from isolated hematuria or proteinuria to rapidly progressive glomerulonephritis with renal failure, without a specific clinical syndrome to facilitate differentiation. In the absence of targeted immunofluorescence and light-chain restriction studies, such as immunohistochemical κ/λ staining to confirm λ-light-chain–restricted deposition, these cases are frequently misdiagnosed as ordinary primary IgA nephropathy. Currently, clinical diagnosis relies primarily on serum protein electrophoresis (SPE), IFE, and serum free light-chain (sFLC) assays. However, SPE and IFE have limited sensitivity and may fail to detect low-level monoclonal proteins, while sFLC testing, despite higher sensitivity, is vulnerable to antigen excess. Renal biopsy with immunofluorescence for κ/λ light chains remains the diagnostic gold standard (17, 18).

Our research group previously described two cases of hypereosinophilic syndrome (HES) associated with elevated IgG4 levels and T-cell clonality, one of which involved IgG4-related interstitial nephritis, ANCA positivity, and multi-organ involvement (19). That study indicated that clonal T-cell receptor rearrangement may drive abnormal immune responses, resulting in immunoglobulin class switching and tissue injury, including renal damage. T cells, particularly follicular helper T cells (Tfh) and regulatory T cells (Treg), regulate B cell differentiation into plasma cells and antibody production via CD40L–CD40 costimulatory interactions, cytokines such as IL-21, and antigen presentation mechanisms (20). In IgA nephropathy and MGRS-associated kidney injury, T cell-mediated immune dysregulation may create a permissive microenvironment for clonal plasma cell expansion, enhance monoclonal IgA-λ production, and mediate renal injury through aberrant glycosylation, immune complex formation, or complement activation, amplifying glomerular inflammation and accelerating disease progression (21). These observations underscore the need for further investigation into the role of clonal immune cell abnormalities, including T cells, B cells, and plasma cells, in MGRS-related multisystem injury.

Here, we describe a case of focal proliferative sclerotic IgA nephropathy that progressed to rapidly progressive nephritis syndrome over two years, gradually involving multiple organ systems. The disease demonstrated poor response to glucocorticoids, conventional immunosuppressants, and anti-CD20 monoclonal antibody therapy. Further evaluation identified a clonal plasma cell abnormality consistent with concurrent IgA-λ monoclonal gammopathy. Upon detection of multisystem injury and monoclonal gammopathy during hospitalization, original kidney pathology slides were re-examined and confirmed via immunoelectron microscopy, which revealed no features of MGRS. The patient later developed pulmonary hemorrhage and neurogenic syncope. High-resolution CT demonstrated diffuse exudative lung lesions without consolidation, cavitation, or nodules. Empirical anti-infective therapy was ineffective, and bronchoalveolar lavage pathogen testing was negative, excluding infectious causes. The pulmonary presentation resembled light chain deposition disease (LCDD), in which monoclonal light chains deposit along alveolar capillary basement membranes, causing hemoptysis. In this patient, monoclonal IgA-λ contained λ light chains that likely deposited along pulmonary vascular basement membranes through a similar mechanism, resulting in vascular injury and alveolar hemorrhage. Pulmonary damage was therefore attributed to monoclonal IgA-λ-mediated vascular injury (22). The patient’s multisystem involvement was considered secondary to IgA-λ monoclonal gammopathy. Lack of response to B-cell-directed therapy but significant improvement with clonal plasma cell-directed therapy further supports that the pathogenic clone originated from plasma cells. The rapidly progressive nephritis and associated renal involvement not only meet the threshold for MGRS but, in the setting of multisystem damage, also fall under the umbrella of MGRhS. Unfortunately, the patient was not eligible for a repeat renal biopsy at that time; therefore, pathological confirmation was lacking.

Bortezomib combined with dexamethasone constitutes first-line therapy for plasma cell–derived MGRS (23, 24) and was employed as a clone-directed treatment in this case. Following three cycles, serum and urine immunofixation became negative, and the serum free light chain ratio normalized, with sustained negativity confirmed on quarterly follow-up. According to International Myeloma Working Group (IMWG) and IKMG consensus guidelines (17, 25), complete hematologic response (CR) is assessed by serologic markers, requiring at least two consecutive negative serum and urine immunofixation tests. These assessments can be done anytime during treatment but before new therapy. Routine repeat bone marrow biopsy is not required for CR confirmation. With sustained serologic negativity and a durable clinical response lasting more than 36 months before transplantation, the patient achieved a clinically significant complete hematologic response. Although dialysis dependence persisted, daily urine output increased, and the frequency of hemodialysis decreased. After three years of maintenance hemodialysis, the patient underwent successful kidney transplantation in April 2022. Post-transplant, the allograft has maintained stable function for three years (most recent follow-up in December 2025), with a normal urinalysis and no monoclonal gammopathy on IFE.

Data on IgAN with monoclonal gammopathy of renal significance (MGRS) in renal transplantation remain limited. Although the post-transplant IgAN recurrence rate ranges from 8% to 53% (26), the risk of MGRS-associated nephropathy recurrence is significantly higher, with the recurrence rate of monoclonal Ig deposition disease exceeding 80% (27). One study showed that the recurrence rate of untreated LCDD reached 89%, while patients who received pre-transplant therapy and achieved remission had a recurrence rate of only 30% (28). These findings indicate that the absence of pre-transplant clone-directed therapy is associated with an increased risk of disease recurrence. Pre-transplant clone-directed therapy and the attainment of a deep hematologic response are critical determinants of reduced recurrence risk. In this case, bortezomib-dexamethasone induced a complete hematologic response before transplantation, which was sustained long-term post-transplant, therefore preserving allograft function and achieving three years of relapse-free survival.

## 4 Conclusion

We report a rare case of IgAN complicated by IgA-λ–type monoclonal gammopathy with multisystem involvement, successfully managed with clonal-directed therapy combined with renal transplantation. When renal pathology in IgAN does not demonstrate MGRS, careful evaluation for monoclonal gammopathy is warranted, as it may cause multi-system damage through MGRhS. Targeted therapy against clonal plasma cells may lead to remission. Clone-directed therapy before transplantation can minimize the risk of recurrence and optimize long-term outcomes.

## Data Availability

The original contributions presented in the study are included in the article/supplementary material. Further inquiries can be directed to the corresponding author.
